# Potency, Efficacy and Durability of DNA/DNA, DNA/Protein and Protein/Protein Based Vaccination Using gp63 Against *Leishmania donovani* in BALB/c Mice

**DOI:** 10.1371/journal.pone.0014644

**Published:** 2011-02-02

**Authors:** Saumyabrata Mazumder, Mithun Maji, Amrita Das, Nahid Ali

**Affiliations:** Infectious Diseases and Immunology Division, Indian Institute of Chemical Biology, Kolkata, India; University of Toronto, Canada

## Abstract

**Background:**

Visceral leishmaniasis (VL) caused by an intracellular protozoan parasite *Leishmania*, is fatal in the absence of treatment. At present there are no effective vaccines against any form of leishmaniasis. Here, we evaluate the potency, efficacy and durability of DNA/DNA, DNA-prime/Protein-boost, and Protein/Protein based vaccination against VL in a susceptible murine model.

**Methods and Findings:**

To compare the potency, efficacy, and durability of DNA, protein and heterologous prime-boost (HPB) vaccination against *Leishmania donovani*, major surface glycoprotein gp63 was cloned into mammalian expression vector pcDNA3.1 for DNA based vaccines. We demonstrated that gp63 DNA based vaccination induced immune responses and conferred protection against challenge infection. However, vaccination with HPB approach showed comparatively enhanced cellular and humoral responses than other regimens and elicited early mixed Th1/Th2 responses before infection. Moreover, challenge with parasites induced polarized Th1 responses with enhanced IFN-γ, IL-12, nitric oxide, IgG2a/IgG1 ratio and reduced IL-4 and IL-10 responses compared to other vaccination strategies. Although, vaccination with gp63 DNA either alone or mixed with CpG- ODN or heterologously prime-boosting with CpG- ODN showed comparable levels of protection at short-term protection study, DNA-prime/Protein-boost in presence of CpG significantly reduced hepatic and splenic parasite load by 10^7^ fold and 10^10^ fold respectively, in long-term study. The extent of protection, obtained in this study has till now not been achieved in long-term protection through HPB approach in susceptible BALB/c model against VL. Interestingly, the HPB regimen also showed marked reduction in the footpad swelling of BALB/c mice against *Leishmania major* infection.

**Conclusion/Significance:**

HPB approach based on gp63 in association with CpG, resulted in robust cellular and humoral responses correlating with durable protection against *L. donovani* challenge till twelve weeks post-vaccination. These results emphasize the potential of DNA-prime/Protein-boost vaccination over DNA/DNA and Protein/Protein based vaccination in maintaining long-term immunity against intracellular pathogen like *Leishmania*.

## Introduction

Leishmaniasis comprise of several diseases caused by a unicellular, digenetic and most genetically diverse protozoan parasites belonging to the genus *Leishmania*. The clinical manifestations range from the self-limiting cutaneous infections to overwhelming visceral disease. There are approximately 1.5 million cases of cutaneous leishmaniasis (CL) and 500,000 visceral leishmaniasis (VL) cases per year [1], [2]. Furthermore, the clinical manifestations of human leishmaniasis depend on the infective parasite species as well as on the host immune response. Current control measures rely on chemotherapy, vector control and control of reservoir host populations. The chemotherapeutic agents used presently are expensive, toxic and emerging drug resistance [3]. For these reasons, reinforced measures for leishmaniasis control, particularly by the development of an affordable and effective vaccine is highly recommended.

Genetic immunization is a relatively new tool for achieving specific immune activation with several advantages such as expression of concerned genes nearest to its native form, induction of cellular immune response, persistent expression of desired antigen (Ag) and induction of memory responses against the infectious disease [4]. Moreover, host cells take up coding plasmids, transcribe and translate the encoded gene, and produce protein that stimulates an immune response when presented to the immune system in the context of self-MHC [5]–[7]. Notably, vaccination with plasmid DNA has been shown to induce protective immunity through both MHC class I- and class II-restricted T cell responses in a variety of infections [8]–[10]. Therefore, the plasmid DNA encoding specific Ag induced both CD4^+^ and CD8^+^ T cells, which is essential for protection against intracellular diseases that require cell mediated immunity like leishmaniasis [11]. Although DNA vaccines have proved effective in the murine system, they are far less protective despite the high doses used in humans [12]–[14]. On the other hand, protein based vaccination induces CD4^+^ T cells probably through MHC-class II restricted pathway resulting in enhanced humoral responses. Despite these advantages, a major limitation in protein vaccination is their inability to elicit strong, durable immune responses when administered alone. Although substantial progress has been obtained toward the effective vaccination strategy, most of the responses they exhibited were short-lived. One promising approach towards the development of potential vaccine candidate in maintaining strong and durable immunity is the “prime-boost” strategy in which immune response is primed with a plasmid DNA and subsequently boosted with either a protein Ag or with a modified viral vector expressing the same Ag [15]. When mice primed with DNA vaccine and later received the same Ag in the form of recombinant protein as boost, production of Th1-type cytokines was increased significantly, as was the IgG2 to IgG1 ratio [15]. In addition, heterologous prime-boost (HPB) vaccination strategy has gained significant momentum against wide ranges of diseases include malaria [16], tuberculosis [17], influenza [18], and HIV [19]. Previous attempts to enhance the protective immune responses against experimental murine leishmaniasis were successful using HPB vaccination strategy [20], [21]. Several leishmanial Ags have been examined as vaccine candidates against murine VL model in prime-boost approach, such as, ORFF [22], CPs [23], and LACK [24]. Moreover, comparative vaccine potential of DNA, protein or HPB vaccination were evaluated against cutaneous form of leishmaniasis in murine model [25]. While all of these studies have resulted in some degree of efficacy, long-term protection (LTP) has rarely been observed. Therefore, in an attempt to examine these findings, to compare the vaccine efficacies and evaluate the durability of such vaccine regimens, we selected gp63 in its DNA and a recombinant form as a vaccine candidate against VL infection.

gp63, a highly conserved protein, is abundantly expressed in promastigotes, and considered the major Ag determinant recognized by the serum samples of patients obtained from different clinical forms of leishmaniasis [26], [27]. Moreover, gp63 has an intrinsic ability to stimulate protective immunity and is a promising vaccine candidate against leishmaniasis. In our previous study, we observed gp63 from *Leishmania donovani* when entrapped within cationic liposomes elicited sustained immune responses against experimental VL [28]. In the present study, we have evaluated the protective and durable immunity raised through gp63 by different vaccination strategies: DNA/DNA, DNA-prime/Protein-boost, Protein/Protein in the susceptible BALB/c mice against experimental VL using CpG-ODN as adjuvant. In addition, we validated the efficiency of the gp63-based vaccine against *Leishmania major* infection in BALB/c mice.

## Materials and Methods

### Animals and parasites

BALB/c mice 4–6 weeks old, reared in the animal care facility under pathogen free conditions were used, for experimental purposes. The studies were approved by the IICB Animal Ethical Committee (147/1999/CPSCEA) and the animals were handled according to their guidelines. *L. donovani* strain AG83 (MHOM/IN/1983/AG83) was maintained by passage in Syrian Hamsters. Promastigotes were cultured in Medium 199 supplemented with penicillin G sodium (100 U/ml), streptomycin sulphate (100 µg/ml) and 10% heat inactivated fetal bovine serum (FBS) (Sigma-aldrich, St. Louis, MO). Parasites from stationary-phase culture were sub-cultured to maintain an average density of 2×10^6^ cells/ml [29]. *L. major* parasites (5ASKH) were grown in Medium 199 supplemented with penicillin G sodium (100 U/ml), streptomycin sulphate (100 µg/ml) and 20% heat inactivated FBS at 22°C.

### Plasmid construction

The gene encoding full-length gp63 of *L. donovani* (GenBank accession number GQ301544) was subcloned from pET16b in frame into pcDNA 3.1 (−/−) (Invitrogen, San Diego, CA) at the BamHI/HindIII restriction sites. The full length gp63 was amplified with gp63-specific primers. The primers used were 5′ CGG GAT CCG GTA TGG GAT CCG TGG ACA GCA GCA GCA CG (forward), and 5′ CCC AAG CTT CTA GAG CGC CAC GGC CAG CAG CGC (reverse) in a Thermocycler (Gene Amp PCR System 9700; Applied Biosystems) using *pfx* Taq DNA polymerase (Invitrogen). PCR conditions were one cycle of 5 min at 94°C, 40 cycles of 1 min at 94°C, 1 min 20 s at 59.5°C, and 2 min at 72°C, followed by a final cycle of 7 min at 72°C. Amplified PCR product was electrophoresed in agarose gel and eluted from the gel (QIA quick gel extraction kit, Qiagen, Valencia, CA). The eluted product was subsequently cloned into mammalian expression vector pcDNA3.1 (−/−) and transformed into competent *Escherichia coli* DH5α cells. The transformants were screened for the presence of recombinant plasmids in presence of ampicillin (Himedia, Mumbai, India). Isolated positive clones were sequenced by DNA sequencer (ABI Prism, Model 377; Applied Biosystems). Recombinant plasmids were then maintained and propagated in DH5α *E*. *coli*. Endotoxin-free plasmid DNA was isolated using Endo-free plasmid isolation kit (Qiagen) and used for *in vitro* transfection and vaccination studies in BALB/c mice.

### Transfection of plasmid constructs and Western blot

CHO-S cells (a gift from Dr. Shiv Sankar Roy) were maintained in RPMI-1640 medium (Invitrogen) supplemented with 10% FBS. The expression of gp63 was detected in mammalian cell by transfecting pcDNA3.1-gp63 construct in CHO-S cell using lipofectamine 2000 (Invitrogen) according to the manufacturer's instructions with slight modifications. Briefly, CHO-S cells were cultured at 1×10^6^ per well in 6-well plates to produce 85–90% confluence on the day of transfection. Lipofectamine 2000 and both pcDNA3.1 vector and pcDNA3.1-gp63 construct were diluted in serum-free Opti-MEM media (invitrogen) at 17 µl/250 µl and 8 µg/250 µl, respectively. The diluted lipofectamine 2000 and plasmid DNA were mixed together and incubated for 25 min at room temperature. The mixture was then added drop wise onto the cell under gentle rocking condition, and incubated for 45 min at room temperature. The transfected cells were incubated 4–6 h at 37°C with 5% CO_2_. 1 ml of RPMI-1640 complemented with 10% FCS was added. The media was replaced 24 h later with fresh media and transfected cells were maintained in presence of 250 µg/ml of G418.

The lysate of stably transfected CHO-S cells was prepared and subjected to SDS-PAGE. Thereafter, the protein bands were electrophoretically transferred to PVDF membrane. To detect the expressed protein, a primary polyclonal antibody against native gp63 [28] was used at 1∶1000 dilution followed by 1∶1000 dilution of HRP-conjugated goat anti-rabbit IgG secondary antibody (Bangalore Genei, Bangalore, India).

### Expression and purification of rgp63

The full-length gp63 was successfully cloned into pET16b vector (Novagen, Madison, USA) previously. For expression of rgp63, *E. coli* BL21 (DE3) pLysS was transformed with pET16bLdgp63 and the construct was grown in 1 L culture medium at 37°C until OD_600 nm_ 0.6 was reached. Protein production was then induced by adding isopropyl β-D-thiogalactoside (IPTG) to a final concentration of 0.5 mM, and incubating for an additional 4 h at 23.5°C. The culture was then harvested by centrifugation at 5,000 *g*, for 5 min, at 4°C, and the cell pellet was resuspended in 6 ml of resuspension buffer (25 mM Tris-HCl, 500 mM NaCl, and 1 mg/ml of Lysozyme, pH 8.0). The cell lysate was sonicated on ice for 5 min with 1 min pulse and 1 min interval between pulses using an ultrasonicator (Misonix, Farmingdale, NY, USA). The sonicated lysate was then centrifuged at 14,000 *g* for 25 min at 4°C and the pellet containing inclusion bodies was solubilised with 5 ml of solublization buffer (25 mM Tris-HCl, 500 mM NaCl, 8 M urea, pH 8.0), kept at room temperature for 30 min and centrifuged at 12,000 *g* for 25 min. The supernatant containing solubilised protein was loaded onto Ni^2+^-nitrilotriacetic acid-agarose (Ni-NTA) column (Qiagen) and purified under denaturing condition. The agarose column was pre equilibriated with equilibriation buffer (25 mM Tris-HCl, 500 mM NaCl, 10 mM imidazole, 8 M urea, pH 8.0). The column was washed with wash buffer (25 mM Tris-HCl, 500 mM NaCl, 50 mM imidazole, 8 M urea, pH 8.0) and eluted with elution buffer (25 mM Tris-HCl, 500 mM NaCl, 500 mM imidazole, 8 M urea, pH 8.0). To refold, the purified materials were diluted 2 fold in dilution buffer containing 25 mM Tris-HCl, 500 mM NaCl, 500 mM imidazole, pH 8.0, and then dialyzed against 25 mM Tris-HCl, 250 mM NaCl, pH 8.0 with decreasing concentration of urea and imidazole. The recombinant proteins were concentrated by Amicon ultrafiltration using a 10-kDa cutoff membrane, exchanged with 25 mM Tris-HCl, 200 mM NaCl, pH 8.0, and finally stored at −70°C. Protein concentrations were determined by Lowry method [30]. Purity and homogeneity of purified proteins was checked by using SDS-PAGE, and the gel was subsequently stained with silver nitrate.

### Immunization of mice and challenge infection

For immunization, BALB/c mice were injected intramuscularly (i.m.) in the hind leg thigh muscle with 50 µg (in 50 µl of PBS) of pcDNA3.1 (−/−) (only vector) or pcDNA3.1-gp63 or PBS. In some groups, CpG-ODN-1826 (20 µg) was used as an adjuvant in combination with plasmid construct. The oligodeoxy nucleotide was synthesized with a nuclease resistant phopshorothioate backbone (Imperial Life Sciences, Haryana, India) and the sequence was 5′ TCC ATG ACG TTC CTG ACG TT 3′. This ODN contained two copies of a CpG motif known to have potent immunostimulatory effects on the murine system [31].

There were two groups in which mice received DNA vaccine either alone (3× gp63 DNA) or in presence of CpG-ODN (3× gp63 DNA+CpG). For immunization with protein, mice were injected through subcutaneous (s.c.) route with 5 µg of rgp63 in combination with 20 µg of CpG-ODN (3× rgp63+CpG). In the heterologous group, mice were primed with two injections of pcDNA3.1-gp63 construct plus CpG, and boosted once with rgp63 plus CpG (2× gp63 DNA+CpG/rgp63+CpG). In some experiments, mice were immunized with either CpG-ODN or rgp63 alone.

For all immunization study, all groups were boosted twice at 2-week intervals. Ten days and twelve weeks after the final booster mice were challenged with 2.5×10^7^ freshly transformed stationary phase *L. donovani* promastigotes in 200 µl PBS injected intravenously as described earlier [29]. For cutaneous infection, 2×10^6^ stationary phase *L. major* promastigotes were injected subcutaneously in the hind footpad. Weekly footpad swelling measurements were recorded using caliper (Starrett Company, Athol, MA).

### Measurement of delayed type hypersensitivity responses (DTH)

DTH response was determined by measuring the difference in the footpad swelling at 24 h following inoculation of the test footpad with 25 µl of rgp63 (200 µg/ml) from that of the control (PBS-injected) footpad with a constant pressure caliper [29].

### Determination of antibody responses

Serum samples of individual mice were obtained before infection, at ten days post vaccination for short-term and twelve weeks post-vaccination for long-term, and after infection, 3 months post challenge for both short and long-term studies. Sera of individual mice were assayed for the presence of gp63-specific IgG1, IgG2a antibodies using enzyme-linked immunosorbent assay (ELISA) as described earlier [29]. In brief, 96-well microtiter plates (Nunc, Naperville, IL) were coated with rgp63 (5 µg/ml) and blocked to prevent nonspecific binding. The plates were then incubated with sera at a 1∶200 dilution, followed by horseradish peroxidase (HRP)-conjugated goat IgG1, and IgG2a (1∶1,000) (BD Pharmingen, San Diego, CA). The color reaction was developed, and the absorbance was read in an ELISA plate reader (Thermo, Waltham, MA) at 450 nm [29].

### Cell proliferation and cytokine assays

Spleens were removed aseptically from experimental mice at the indicated time before and after infection, and the single cell suspensions were prepared in RPMI-1640 supplemented with penicillin G sodium (100 U/ml), streptomycin sulphate (100 µg/ml) and 10% heat inactivated FBS and 50 µM mercaptoethanol (Sigma-Aldrich, St. Loius, MO). Erythrocytes were removed by lysis with 0.14 M Tris-buffered NH_4_Cl. The splenocytes were washed twice and resuspended in the culture medium, and viable mononuclear cell numbers were determined by trypan blue exclusion [32]. The cells were then cultured in triplicate in a 96 well flat bottom plate (Nunc, Roskilde, Denmark) at a density of 2×10^5^ cells/well in a final volume of 200 µl complete medium and stimulated with rgp63 (5 µg/ml). Cells were incubated at 37°C in a humified chamber containing 5% CO_2_. For cytokine analysis, cells were stimulated for 96 h, and supernatants were collected and the concentrations of of IFN-γ, IL-4, IL-12(p40) and IL-10 (BD Pharmingen,) were quantitated by ELISA in accordance with the manufacturer's instructions [32].

For blocking experiments, aliquots of viable splenocyte cells were incubated with anti-CD4 and anti-CD8 monoclonal antibodies (mAbs) or the respective control IgGs for 1 h at 4°C and washed twice in complete medium. The efficiency of blocking was checked by flow cytometry. Almost 93% of CD4^+^ and 75% of CD8^+^ T cells were blocked through this procedure. Total, and CD4- or CD8-blocked splenocytes [33] were stimulated *in vitro* with medium alone or with rgp63 (5 µg/ml) for 96 h.

For cell proliferation assay, the cells were incubated for 96 h and pulsed with 1 µCi of [^3^H]-Thymidine (Amersham Biosciences, Buckinghamshire, UK) per well 18 h before they were harvested on glass fiber paper. Thymidine uptake was measured in a β-scintillation counter (Beckman Instruments, Fullerton, CA) [34].

### Measurement of NO production

Nitric oxide (NO) levels, quantified by the accumulation of nitrite in the culture medium, were measured as described previously [32]. Briefly, 100 µl of splenocyte culture supernatants were mixed with an equal volume of Griess reagent (1% sulfanilamide and 0.1% *N*-1-naphthylethylene diamine hydrochloride in 50% H_3_PO_4_) and incubated at room temperature for 10 min. Absorbance was then measured at 540 nm.

### Evaluation of parasite burden in liver and spleen

Following 3 months post-challenge infection in both short-and long-term studies, parasite load was evaluated by limiting dilution assay (LDA) with slight modifications [35]. Briefly, a weighted piece of liver or spleen from an experimental mouse was homogenized in complete Schneider's Drosophila medium (Invitrogen, Grand island, USA) containing 10% heat inactivated FBS and diluted with the same medium to a final concentration of 1 mg/ml. Five-fold serial dilutions of homogenized tissue suspensions were then plated in a 96-well tissue culture plates (Nunc, Roskilde, Denmark) and were cultured for one month at 22°C. Wells were examined for viable and motile promastigotes at 5 day- interval, and the reciprocal of the highest dilution that was positive for parasites was considered to be the concentration of parasites per mg of tissue. The total organ burden was calculated using the weight of the respective organs.

### Statistical analysis

One-way ANOVA analysis (Multiple comparisons Tukey's post hoc test) was performed using the GraphPad InStat software. A value of *p*<0.05 was considered to be significant.

## Results

### Construction of full-length gp63 gene in mammalian pcDNA3.1 (−/−) expression vector, its expression into CHO cell line, and expression and purification of rgp63 in *E. coli* strain

**Figure 1:**
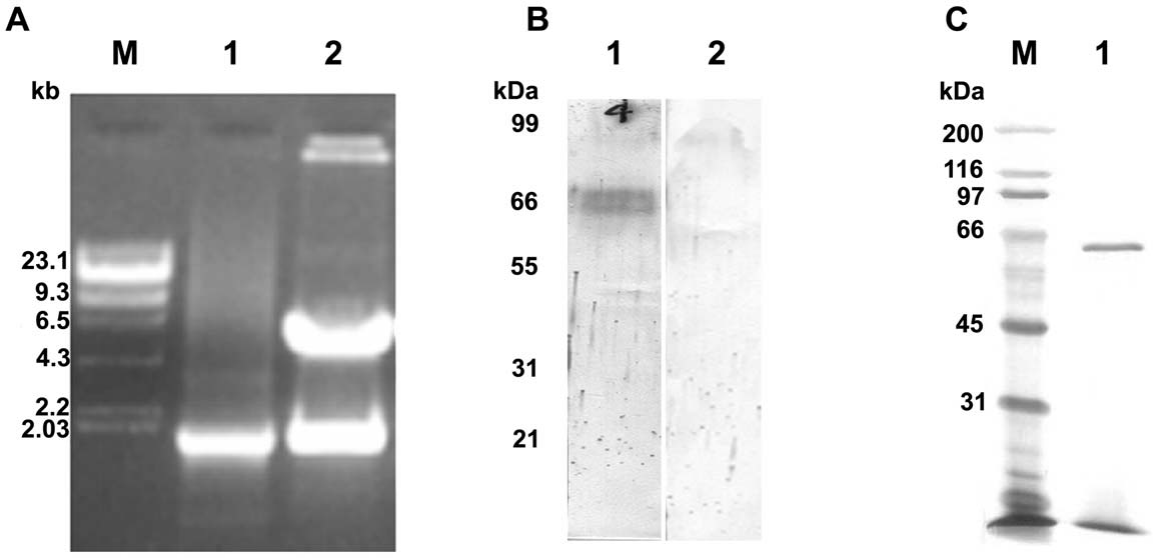
Cloning and expression of *L. donovani* gp63 in mammalian expression vector and purification from *E. coli*. (A) Clone confirmation of gp63 in pcDNA3.1 (−/−) vector. M, λDNA digested with HindIII marker; lane 1, PCR of cloned construct; lane 2, BamHI/HindIII digested pcDNA3.1-gp63 construct. (B) Expression of gp63 in transfected CHO cell line. Lane 1, western blot of pcDNA3.1-gp63 transfected construct in CHO cell line; lane 2, western blot of pcDNA3.1 transfected vector in CHO cell line. (C) Silver nitrate staining of 10% SDS-PAGE, M, molecular mass marker; lane 1, Purified recombinant gp63.

Full length GP63 was successfully subcloned in right orientation under the mammalian expression vector pcDNA3.1 (−/−). The positive clones were selected by using PCR and restriction enzyme digestion analysis (Figure 1A), and the PCR product was further sent for sequence analysis. The recombinant plasmids were then transfected into the CHO cell line and the expression at the protein level was confirmed by western blot analysis using anti-gp63 antibody raised in rabbits (Figure 1B). The results showed that the recombinants were correctly constructed and could be expressed in mammalian cell line.

The over expressed protein from *E. coli* BL21 (DE3) pLysS cells harboring plasmid pET16bLdgp63 was purified through Ni^+2^-NTA agarose column under denaturing conditions. The recombinant protein was refolded, dialyzed and finally concentrated using Amicon ultrafiltration 10-kDa cut-off membrane. The yield of purified protein was approximately 0.5 mg per liter of culture. Analysis of the purified rgp63 showed that the protein was essentially homogeneous (Figure 1C).

### Humoral responses

Since the outcome of VL may be determined by the extent of immune system activation, it was highly important to characterize the changes in the immunoglobulin ratios after immunization. It is well established that the cytokines such as IFN-γ and IL-4 direct immunoglobulin class switching of IgG2a and IgG1, respectively. We therefore analyzed rgp63-specific production of these antibodies before infection. Although very low levels of IgG2a and IgG1 titers in all vaccinated group was observed, sera from mice immunized with HPB regimen showed significantly enhanced IgG2a (*p*<0.001), a surrogate marker for Th1, (Table 1) in comparison to gp63 DNA vaccinated groups in short-term study. This humoral response was maintained till twelve weeks after vaccination, and reached levels that were significantly higher than groups of mice receiving rgp63 along with CpG (Table 2) (*p*<0.001). Moreover, IgG1, a surrogate marker for Th2 cell differentiation, was elevated particularly in HPB vaccine groups and mice receiving rgp63 and CpG in both short-and long-term studies (Table 1 and 2). Therefore, mice vaccinated either heterologously or homologously using protein based vaccination with CpG- ODN were able to elicit mixed Th1/Th2 responses before infection.

**Table 1:** Ag-specific IgG isotype responses in mice vaccinated with different vaccine regimens in short-term protected group (ten days post boost) before and 3 months after *L. donovani* challenge infection.

Time points	Vaccination groups (Short-term protection)	Antibody titer (O.D at 450 nm)		
		Serum IgG2a	Serum IgG1	Ratio of serum IgG2a/IgG1
Before infection	PBS	0.089±0.1	0.096±0.01	0.926±0.04
	Only Vector	0.095±0. 0	0.101±0.01	0.940±0.05
	3× gp63 DNA	0.183±0.01	0.123±0.01	1.531±0.22
	3× gp63 DNA + CpG	0.205±0. 17	0.144±0.01	1.340±0.14
	2× gp63 DNA + CpG/rgp63 + CpG	0.3±0. 01^a^	0.180±0.02	1.765±0.28
	3× rgp63 + CpG	0.252±0.02	0.167±0.01	1.538±0.27
Post-infection	PBS	0.147±0.02	0.135±0.02	0.922±0.29
	Only Vector	0.127±0.01	0.137±0.02	0.983±0.14
	3× gp63 DNA	0.208±0.01	0.107±0.01	2.042±0.27
	3× gp63 DNA + CpG	0.268±0.01	0.118±0.01	2.365±0.18
	2× gp63 DNA + CpG/rgp63 + CpG	0.343±0.02	0.111±0.0	3.176±0.24^c^
	3× rgp63 + CpG	0.575±0.02^b^	0.258±0.01^b^	2.244±0.16

Ten days after final immunization and 3 months after challenge infection, blood serum samples were collected and assayed for IgG2a, IgG1, and IgG2a:IgG1 by ELISA. The results are shown as the mean absorbance values ± S.E. of five individual mice per group, representative of two independent experiments with similar results. *p* values were calculated using one-way ANOVA and Tukey's multiple comparison test.

a Significantly higher than gp63 DNA either free or in presence of CpG (*p*<0.001).

b Significantly higher than DNA-prime/Protein-boost (*p*<0.001).

c Significantly higher than gp63 DNA (*p*<0.05).

Next, we investigated IgG2a and IgG1 titers in all the vaccinated mice following challenge with *L. donovani*. After challenge infection, although mice immunized with rgp63 plus CpG-ODN showed enhanced IgG2a and IgG1 titers, mice vaccinated with either gp63 DNA alone or in association with CpG-ODN or DNA-prime/Protein-boost slightly induced the levels of IgG2a and IgG1 (Table 1 and 2). In the LTP study, twelve weeks after final vaccination and at 3 months post-infection, only gp63 DNA immunization increased IgG2a titer by 2.3 fold and incorporation of CpG motifs to the gp63 DNA vaccination increased the titer by 2.56 fold (Table 2). Most surprisingly, almost 4.22 fold IgG2a titer was obtained in mice receiving rgp63 along with CpG. Although protein based vaccination induced substantial IgG2a responses after challenge infection, the IgG2a/IgG1 ratio before and after infection in mice vaccinated with rgp63 plus CpG and DNA-prime/Protein boost were 1.18±0.04 and 3.01±0.34 and 1.534±0.085 and 4.562±0.74 respectively, in long-term experiments (Table 2).

**Table 2:** Ag-specific IgG isotype responses in mice vaccinated with different vaccine regimens in long-term protected group (twelve weeks post boost) before and after 3 months *L. donovani* challenge infection.

Time points	Vaccination groups (Long-term protection)	Antibody titer (O.D at 450 nm)		
		Serum IgG2a	Serum IgG1	Ratio of serum IgG2a/IgG1
Before infection	PBS	0.084±0.01	0.096±0.01	0.877±0.06
	OV	0.097±0.0	0.105±0.0	0.760±0.17
	3× gp63 DNA	0.147±0.01	0.123±0.01	1.193±0.07
	3× gp63 DNA + CpG	0.177±0.01	0.139±0.01	1.282±0.01
	2× gp63 DNA + CpG/rgp63 + CpG	0.309±0.018^b^	0.202±0.01^a^	1.534±0.08
	3× rgp63 + CpG	0.221±0.01	0.186±0.0	1.184±0.04
Post-infection	PBS	0.119±0.0	0.165±0.01	0.654±0.14
	OV	0.128±0.0	0.175±0.0	0.731±0.03
	3× gp63 DNA	0.304±0.01	0.103±0.01	3.061±0.32
	3× gp63 DNA + CpG	0.456±0.05	0.128±0.02	3.710±0.44
	2× gp63 DNA + CpG/rgp63 + CpG	0.548±0.03	0.133±0.02	4.562±0.74
	3× rgp63 + CpG	0.935±0.04^c^	0.333±0.05^c^	3.011±0.34

Twelve weeks after final immunization and 3 months after challenge infection, blood serum samples were collected and assayed for IgG2a, IgG1, and IgG2a:IgG1 by ELISA. The results are shown as the mean absorbance values ± S.E. of five individual mice per group, representative of two independent experiments with similar results. *p* values were calculated using one-way ANOVA and Tukey's multiple comparison test.

a Significantly higher than gp63 DNA either free or in presence of CpG (*p*<0.001).

b Significantly higher than gp63 DNA either alone or in association with CpG, and rgp63 plus CpG (*p*<0.001).

c Significantly higher than DNA-prime/Protein-boost (*p*<0.001).

### Delayed type hypersensitivity and splenocyte proliferation

To verify the generation of cellular immune responses in DNA/DNA, DNA/Protein and Protein/Protein based vaccination against experimental VL, we used gp63 vaccine in its various forms in association with a TLR9 agonist, CpG-ODN.

**Figure 2:**
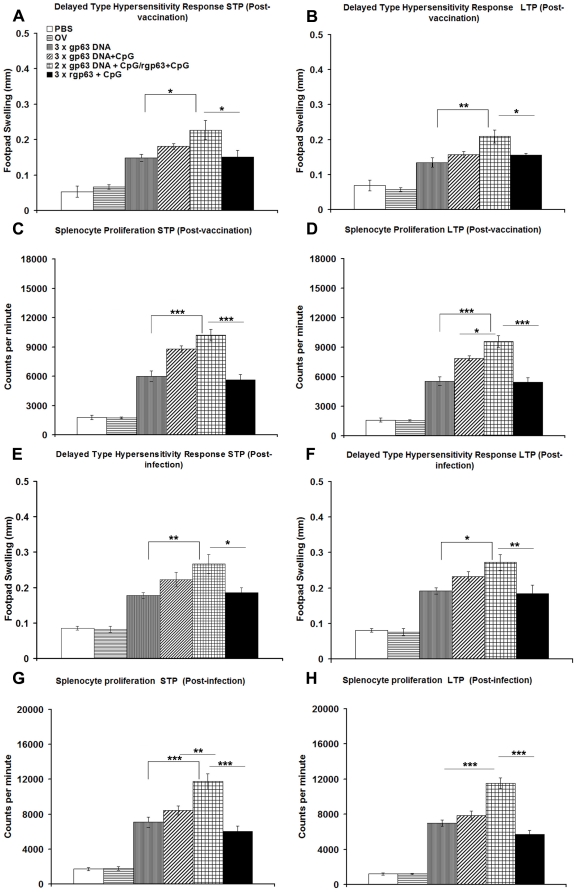
DTH and splenocyte proliferation in mice vaccinated with gp63 with different vaccination approaches before and after 3 months challenge infection. Ten days, short-term protection (STP), and twelve weeks, long-term protection (LTP), after final boosting (post-vaccination) (A–D), and 3 months after challenge infection (post-infection) (E–H) rgp63-specific DTH and splenocyte proliferation was measured. DTH response was determined by measuring the difference in the footpad swelling at 24 h following inoculation of the test footpad with 25 µl of rgp63 (200 µg/ml) from that of the control (PBS-injected) footpad. Spleens were collected and splenocytes were re-stimulated *in vitro* for 96 h with 5 µg/ml of rgp63 and pulsed with 1 µCi of [^3^H]-Thymidine for 18 h. Ag-specific splenocyte proliferation was determined by Thymidine incorporation and expressed as counts per minute. Figures (A, B, E, F) represent DTH and (C, D, G, H) splenocyte proliferation in STP and LTP studies. The results are shown as the mean values ± S.E. of five individual mice per group, representative of two independent experiments with similar results. OV- only vector. * *p*<0.05, ** *p*<0.01, *** *p*<0.001 as assessed by one-way ANOVA and Tukey's multiple comparison test.

DTH, an index of cell mediated immunity *in vivo*, and an Ag-specific *in vitro* T cell proliferation assay revealed the status of cellular responses generated in vaccinated animals. We were therefore interested to see the DTH and proliferative responses elicited by vaccinated and challenged animals. BALB/c mice immunized heterologously showed significantly higher DTH responses compared to mice vaccinated with gp63 DNA alone or rgp63 mixed with CpG (*p*<0.05) before infection in both short-as well as long-term experiments (Figure 2A and B). Moreover, Ag-specific proliferative responses elicited by HPB vaccination regimen was significantly (*p*<0.001) higher in comparison to mice vaccinated with only gp63 DNA and protein plus CpG vaccinated group in both types of protection study (Figure 2C and D). The specificity of the responses after gp63 vaccination was tested using gp63-non related protein such as Cysteine Protease A (CPA) of *L. donovani*. Inoculation of test footpad of BALB/c mice or *in vitro* splenocytes pulsing with recombinant CPA (rCPA) confirmed that the responses generated herein were specific to gp63 and not to other gp63 non-related protein, rCPA (data not shown).

Challenge with *L. donovani* induced enhanced DTH responses and rgp63-specific T cell proliferation in all vaccinated mice. Among the different vaccinated groups, mice receiving heterologous immunization exhibited the highest degree of both DTH and proliferative response of splenocytes compared to mice vaccinated with gp63 DNA alone or rgp63 in association with CpG in short-term protection (STP) study (Figure 2E and G).

In the long-term, the proliferative responses of splenocytes from heterologously vaccinated mice increased from 9600±578.8 cpm to 11546±610.8 cpm after challenge infection suggesting that DNA-prime/Protein-boost regimen induced long-term cellular responses compared to all vaccinated mice (*p*<0.001) (Figure 2D and H). Therefore, vaccination with DNA-Prime/Protein-boost showed highest Ag-specific DTH and proliferative responses in comparison to either DNA/DNA or Protein/Protein vaccination before and after *L. donovani* challenge infection.

### Cytokine responses

It is well established that the cytokine milieu at the initiation of infection is critical in determining disease outcome [36]-[37]. So to understand the interplay between the disease healing inflammatory cytokines IFN-γ, and IL-12 and disease associated cytokines IL-10 and IL-4, we sought to investigate Ag-specific *in vitro* production of cytokines before and after challenge infection in both STP and LTP studies.

**Figure 3:**
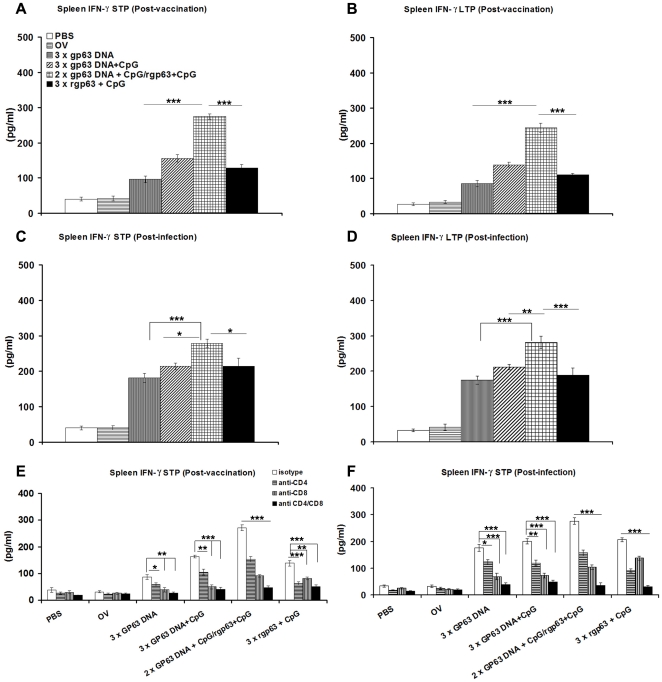
IFN-γ responses in BALB/c mice vaccinated with different vaccine approaches before and after 3 months challenge infection. Levels of IFN-γ ten days, short-term protection (STP), and twelve weeks, long-term protection (LTP) after final boosting (post-vaccination) (A, B), and 3 months after challenge infection (post-infection) (C, D). Splenocytes were isolated from vaccinated mice, stimulated with rgp63 (5 µg/ml) and were cultured for 96 h. The supernatants were collected, and assayed for IFN-γ through ELISA. Figures (E, F) represent *in vitro* blocking experiments either with anti-CD4^+^ or anti-CD8^+^ or both mAbs before (post-vaccination) and after *L. donovani* infection (post-infection). The results are shown as the mean absorbance values ± S.E. of five individual mice per group, representative of two independent experiments with similar results. OV- only vector. * *p*<0.05, ** *p*<0.01, *** *p*<0.001 as assessed by one-way ANOVA and Tukey's multiple comparison test.

Mice immunized with different forms of gp63 vaccines induced IFN-γ before infection (Figure 3A and B). Addition of CpG to gp63DNA resulted in enhancement of IFN-γ production from (96.6±9.516) pg/ml to (155.8±10.92) pg/ml in STP study (Figure 3A). Therefore, addition of CpG to gp63 DNA skewed the response towards Th1 type. Furthermore, HPB regimen secreted significantly higher levels of IFN-γ (275±7.8 pg/ml) in comparison to mice receiving rgp63 in association with CpG or gp63 DNA either alone or in combination with CpG. These responses were almost maintained after twelve weeks post-vaccination (Figure 3B). The IFN-γ responses in splenocytes measured after *L. donovani* challenge also demonstrated that there was a higher IFN-γ for DNA-prime/Protein-boost vaccination than in others (Figure 3C and D). Therefore, the *in vitro* restimulation with rgp63 at twelve weeks after booster vaccination demonstrated that there was induction of IFN-γ responses in HPB regimen more significantly compared to other vaccinated strategies. We then analyzed, the contribution of CD4^+^ and CD8^+^ T cells to the rgp63-specific production of IFN-γ *in vitro*. As shown in figure (3E and F) cytokine synthesis induced by rgp63 in DNA based vaccine was mainly inhibited by mAb to CD8^+^ T cells and partially inhibited by anti-CD4^+^ mAb. In contrast, production of IFN-γ mainly inhibited by anti-CD4^+^ Ab and partially by anti-CD8^+^ Abs in mice vaccinated with rgp63 along with CpG. However, in DNA-prime/Protein-boost based vaccination, contribution of both these cell types was involved.

**Figure 4:**
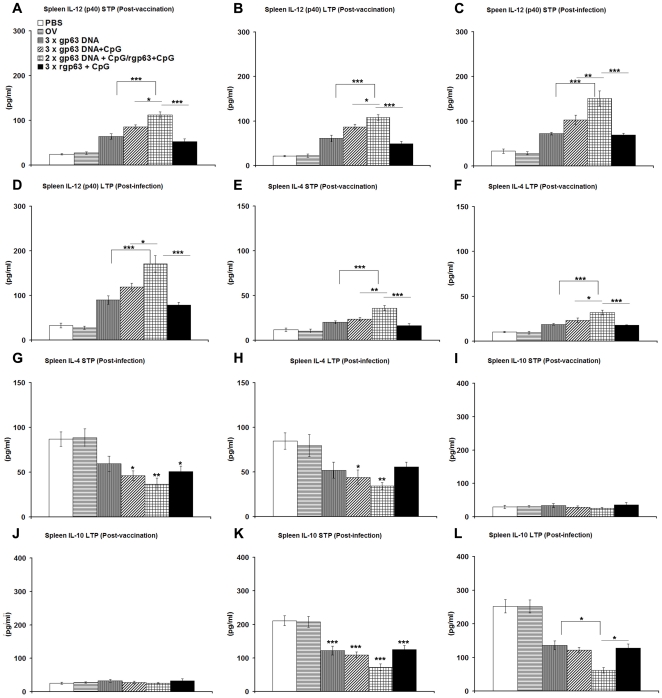
IL-12, IL-4 and IL-10 responses in BALB/c mice vaccinated with different vaccine regimens before and after 3 months challenge infection. Ten days, short-term protection (STP), and twelve weeks, long-term protection (LTP) after final boosting (post-vaccination), and 3 months after challenge infection (post-infection) splenocytes were collected from vaccinated mice, stimulated with rgp63 (5 µg/ml) and were cultured for 96 h. The supernatants were collected, and assayed for IL-12 (A–D), IL-4 (E–H), and IL-10 (I–L) through ELISA. The results are shown as the mean absorbance values ± S.E. of five individual mice per group, representative of two independent experiments with similar results. OV- only vector. * *p*<0.05, ** *p*<0.01, *** *p*<0.001 as assessed by one-way ANOVA and Tukey's multiple comparison test.

Similar pattern was observed also in IL-12 production (Figure 4A-D). Highest IL-12 production was observed in mice vaccinated with DNA-prime/Protein-boost group, which was significantly higher in mice vaccinated with either gp63 alone (*p*<0.001), or in combination with CpG (p<0.05), or rgp63 in association with CpG (*p*<0.001) in both short and long-term studies before challenge infection (Figure 4A and B). After challenge infection, the level of IL-12 was enhanced further in all vaccinated groups of mice in both STP and LTP experiments (Figure 4C and D) and the level of IL-12 production in HPB regimen was significantly higher than all the other vaccinated groups. Moreover, HPB vaccination increased IL-12 levels by 1.34 fold and 1.58 fold in both short-and long-term protected group after *Leishmania* challenge.

However, before and after infection, the levels of IL-4, an established Th2 cytokine, was significantly higher in mice vaccinated heterologously compared to mice receiving gp63 DNA alone (*p*<0.001), or in association with CpG (p<0.01) and rgp63 plus CpG (*p*<0.001) (Figure 4E and F). Conversely, level of expression of Th1 suppressive cytokine, IL-10 was unaffected in vaccinated mice compared to controls (Figure 4I and J).

At 3 months post-infection, both STP and LTP studies revealed that mice vaccinated with HPB vaccination could significantly down regulate IL-4 in comparison to controls (*p*<0.01) (Figure 4G and H). In case of challenge infection, HPB regimen showed almost 2.35 fold and 2.28 fold reductions in IL-4 in STP and LTP studies respectively compared to control PBS. Similarly, mice prophylactically immunized with DNA-prime/Protein-boost resulted in 2.56 fold and 3.5 fold decreased IL-10 secretion compared to control after *L. donovani* infection in both short as well as long-term studies respectively. Furthermore, down regulation of IL-10 production from splenocytes of HPB regimen was significantly lower than group of mice receiving rgp63 plus CpG or gp63 DNA alone, in the long-term (*p*<0.05) (Figure 4L). Hence the results demonstrate generation of early mixed Th1/Th2 responses before infection, followed by strong Th1 biased response in mice receiving DNA-prime/Protein-boost vaccine after *L. donovani* challenge. Therefore, the vaccination strategies employed with either DNA/DNA or Protein/Protein in presence or absence of CpG have resulted weaker cellular responses than DNA-prime/Protein-boost before and after challenge infection.

### Measurement of NO

**Figure 5:**
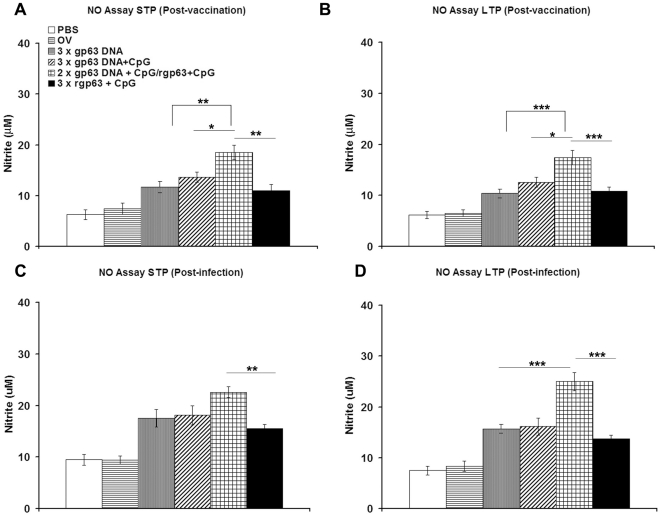
Ag-specific NO levels in vaccinated mice before and after 3 months challenge infection. Ten days, short-term protection (STP), and twelve weeks, long-term protection (LTP) after final boosting (post-vaccination), and 3 months after challenge infection (post-infection), splenocytes from different vaccinated mice were isolated and stimulated with rgp63 (5 µg/ml) for 96 h and the level of NO was determined in supernatants by Griess reagent. Figures (A, B) and (C, D) represent levels of NO before (post-vaccination) and after 3 months of *L. donovani* infection (post-infection) respectively. The results are shown as the mean absorbance values ± S.E. of five individual mice per group, representative of two independent experiments with similar results. OV- only vector. * *p*<0.05, ** *p*<0.01, *** *p*<0.001 as assessed by one-way ANOVA and Tukey's multiple comparison test.

NO is the critical killing effector molecule against leishmaniasis produced by IFN-γ stimulated and inducible NO synthase-induced classical macrophages. To evaluate the killing effector functions of prophylactic DNA/DNA, DNA-prime/Protein-boost, Protein/Protein based approach in vaccinated and challenged mice, NO was determined from splenocyte culture supernatant. Spleen cells from PBS, only vector, gp63 DNA, gp63 DNA mixed with CpG, HPB regimen, or rgp63 mixed with CpG-immunized mice before and after infection in both short as well as long-term protected groups were stimulated with rgp63 and supernatants were tested for NO assay (Figure 5). We found that HPB regimen showed considerably highest (18.5 and 17.4 µM) nitrite production in short and long-term studies, before challenge infection. The response was significantly higher than mice vaccinated with either gp63 DNA alone (*p*<0.001), or gp63 DNA mixed with CpG (*p*<0.05), and rgp63 associated with CpG (*p*<0.001) (Figure 5B) in the long-term study. Moreover, after challenge infection, the HPB regimen showed 22.6 and 25 µM nitrite production in both short and long-term studies respectively. HPB regimen, therefore showed highest magnitude of NO production which was significantly higher than other vaccinated groups of mice (*p*<0.001) (Figure 5C and D) and maintained till twelve weeks post-vaccinated mice receiving *L. donovani* challenge infection.

### Determination of splenic and liver parasite load following challenge infection with *L. donovani*

**Figure 6:**
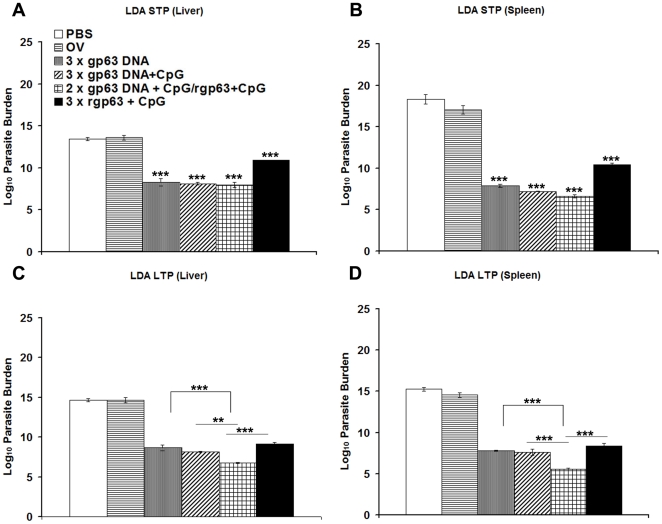
Evaluation of protection against *L. donovani* challenge in mice vaccinated with different vaccine regimens. Quantification of single viable cell was carried out by limiting dilution assay performed 3 months after infection on cells isolated from liver (A, C) and spleen (B, D) in ten days, short-term protection (STP) (A, B) and twelve weeks, long-term protection (LTP) (C, D) studies. The cells were cultured in duplicate in complete Schneider's Drosophila medium containing 10% FCS for 1 month at 22°C in serial five-fold dilutions. The reciprocal of the highest dilution that was positive for parasite growth was considered to be the concentration of parasites per mg of tissue. Results were expressed as log of total organ parasite burden. Data represent the mean ± S.E of five individual mice per group of one experiment. OV- only vector. ** *p*<0.01, *** *p*<0.001 as assessed by one-way ANOVA and Tukey's multiple comparison test. *** *p*<0.001 in comparison to controls unless stated.

Because HPB regimen showed impressive humoral and cellular immune responses in comparison to other vaccinated groups, we were interested to check the prophylactic efficacies of DNA/DNA, DNA-prime/Protein-boost and Protein/Protein vaccines on the clearance of splenic and hepatic parasite burden following challenge infection. Fig. 6 illustrates the outcome of challenge infection in BALB/c mice challenged either after ten days and twelve weeks after last boosting and progression of infection was monitored at 3 months. The degree of protection was quantified in liver and spleen through serial dilution assay, which is more reliable for monitoring low parasite loads as well as viable parasites. Mice vaccinated with different vaccine formulations with gp63 DNA showed comparable levels (almost 10^5–6^ fold reductions compared to PBS control, *p*<0.001) of protection against the development of parasite burden in the liver of STP study (Figure 6A). However, only 10^3^ fold reduction (compared to PBS, *p*<0.001) in hepatic parasite load was achieved in mice vaccinated with rgp63 and CpG. Surprisingly, the parasite clearance in the spleen was efficiently achieved by all the vaccinated mice in STP study (Figure 6B). Mice vaccinated with different gp63 DNA vaccine regimens showed comparable levels (10^11^ fold reductions in parasite load compared to PBS, *p*<0.001) of parasite clearance in spleen. However, almost 10^7^-fold reduction in parasite load was also obtained in mice vaccinated with rgp63 mixed CpG, compared to control saline (*p*<0.001).

To evaluate the durability of the immunity induced by DNA/DNA, DNA-prime/Protein-boost and Protein/Protein vaccines, the length of protection was determined by examining the responses to challenge infection at twelve weeks after booster vaccination, sacrificed 3 months post-infection, and the parasite load was again determined through serial dilution. Our data demonstrate that, mice vaccinated heterologously showed significantly higher reduction in hepatic parasite load (almost 10^7^ fold compared to control PBS, *p*<0.001) in comparison to either only gp63 DNA (*p*<0.001), or DNA mixed with CpG (*p*<0.01) and rgp63 mixed with CpG (*p*<0.001) (Figure 6C). Similar responses were observed in clearing parasites in spleen. HPB regimen showed highest reduction in parasite load (almost 10^10^ fold reduction compared to PBS, *p*<0.001) in comparison to gp63 DNA either alone or in association with CpG (*p*<0.001) or rgp63 mixed with CpG (*p*<0.001) (Figure 6D). Interestingly, mice vaccinated heterologously, showed a hepatic parasite burden of 7.9 log_10_±0.31 in short-term and 6.75 log_10_±0.06 in long-term and splenic parasite burden of 6.26 log_10_±2.00 in short-term and 5.42 log_10_±0.158 in LTP studies. Therefore, in terms of organ parasite burdens, the immunity conferred by priming twice with gp63 DNA mixed with CpG, followed by single boosting with rgp63 mixed with CpG was more effective and its potency and durability was maintained till twelve weeks.

**Figure 7:**
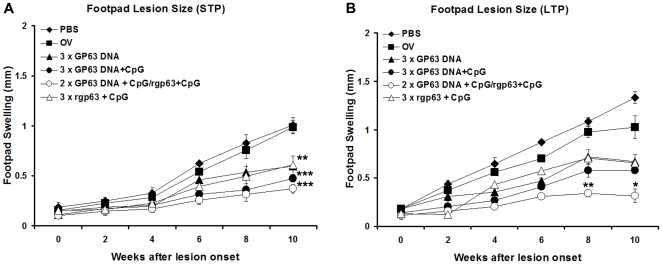
Course of *L. major* infection in gp63 vaccinated BALB/c mice. Ten days, short-term protection (STP) study (A), and twelve weeks, long-term protection (LTP) study (B), after last immunization, mice were challenged with 2×10^6^ *L*. *major* through s.c. route in the hind footpad. Lesion development was monitored by measurement of footpad thickness for 10 weeks. Each point represents the average increase in footpad thickness ± S.E of four individual mice per group. The experiment has been performed once. OV- only vector. **p<0.01, ***p<0.001 compared to controls (A). *p<0.05 and **p<0.01 significantly differs from mice vaccinated with gp63 DNA, rgp63 plus CpG respectively (B).

Since HPB regimen showed durable immune responses against *L. donovani* infection, we were interested to check the vaccine efficacy of different gp63-based vaccination against CL. Mice immunized with heterologously or rgp63 based vaccination induced significantly smaller lesion size compared to PBS (*p*<0.001, *p*<0.01) in STP (Figure 7A). Furthermore, in the LTP study, all mice receiving gp63-based vaccination showed reduced lesion size compared to PBS (*p*<0.001) (Figure 7B). Most interestingly, vaccination with DNA-prime/Protein-boost showed significantly lesser lesion sizes in comparison to the gp63 DNA (*p*<0.05) or rgp63 plus CpG (*p*<0.01). Hence, the HPB approach using *L. donovani* gp63 also validates its protective role against *L. major* infection in BALB/c mice.

## Discussion

In this study, we evaluate the comparative vaccine potential of DNA/DNA, DNA-prime/Protein-boost, and Protein/Protein based vaccination using gp63 with a toll-like receptor ligand (TLR-9) agonist CpG-ODN in eliciting short- as well as long-lasting immunity against infections with *L. donovani* in genetically susceptible BALB/c mice. The results reported here suggest that the control of infection was effectively achieved by DNA-prime/Protein-boost based vaccination in a sustained manner. However, the immunity conferred by DNA/DNA or Protein/Protein vaccination was less effective particularly during long-lasting study, while Protein/Protein-based vaccination induced partial protection during short-term experiments. Since the goal of any vaccine is to generate a sustain immune response over a long period of time, we explored the effectivity of DNA-prime/Protein-boost immunization with an immunomodulator, CpG-ODN over DNA/DNA and Protein/Protein vaccination in LTP against VL using gp63 as candidate vaccine.

Previous studies using gp63 DNA have been shown strong Th1 biased responses with varying levels of protection in mice against the cutaneous form of the leishmaniasis [10], [38]–[41]. Moreover, with regard to *L. donovani* infection, genetic immunization with gp63 either in DNA form or through HPB approach with rgp63 induced Th1 biased response, while protein boosting did not significantly affect the efficacy of vaccines in terms of hepatic and splenic parasite load [42]. Therefore, to evaluate the potency, efficacy and durability, a comparative study using either genetic or heterologous or protein-based vaccination with gp63 is required against VL. Since successful vaccination in VL was associated with an initial mixed Th1/Th2 response after immunization and with challenge infection the response polarized towards Th1 with further boosting of IFN-γ and down regulation of IL-4 [32], we demonstrate, DNA-prime/Protein-boost vaccination strategy significantly induced mixed Th1/Th2 responses after immunization, while *L. donovani* challenge infection induced significantly strong Th1 biased response compared to other vaccination strategies. The difference we observed in our work with the published report [42] could be due to the use of CpG-ODN in our formulations. The results we obtained from our study is the first report, so far as we are aware, of using different vaccination strategies with gp63 from *L. donovani* in short as well as long-lasting protection against experimental VL.

A primary goal of vaccination is to induce memory responses that will provide long-lived protection against severe disease by intracellular pathogen. Despite maintaining sustained long-lasting immunity through DNA vaccination against cutaneous form of leishmaniasis [9], [43], the reduction in parasite load was almost 10^3–4^ folds depending on the types of tissue examined. Moreover, our previous study with native gp63 demonstrated that liposomal delivery of protein Ag conferred long-term protection was in contrast to previous studies [33], [43]. However, this result was obtained through intraperitoneal (i.p.) immunization, a route that was not desired for human vaccination [28]. Therefore, effective vaccine strategies are still required for elicitation of immune responses and durability against *Leishmania* infection.

Effective and successful vaccinations against intracellular pathogens require persuasive adjuvant that can induce strong immune response. The adjuvant like properties of CpG-ODN on protein-based vaccination was observed particularly in maintaining long-lasting immunity [44] which overcome the need for IL-12 in genetically susceptible BALB/c mice infected with *L. major*. In addition, although, unmethylated CpG motifs within the plasmid DNA vector have also been shown to contribute to the immunogenicity of DNA vaccines [45], [46], cloning additional CpG motifs or adding CpG-ODN to the DNA vaccine rendered substantial immune responses [47], [48]. Therefore, using CpG-ODN was thought to be effective regarding long-lasting protection against VL.

The nature of immune responses in vaccinated mice was determined by the level of IgG2a and IgG1 antibody isotypes in sera that associated with Th1 and Th2 response, respectively. We observed mice immunized with HPB regimen showed early mixed Th1/Th2 response that could lead to successful vaccination after challenge. Induction of the anti-*Leishmania* IgG2a, and unaltered IgG1 after infection in DNA vaccinated group, suggested polarized Th1 responses. By contrast, conventional protein based vaccine elicited enhanced *Leishmania* specific IgG2a and IgG1 antibody levels, particularly when combined with CpG ODN after infection. Furthermore, *L. donovani* infection in HPB regimen induced higher IgG2a/IgG1 ratio suggesting its strong ability towards protective Th1 biased response.

Because the cell mediated immune response is severely affected during active VL due to T cell anergy specific to *Leishmania* Ags [49], [50], successful vaccination of humans and animals is often related to Ag-induced DTH responses *in vivo* and T cell proliferation *in vitro* [51]. We observed that vaccination heterologously resulted in an enhanced DTH and splenocyte proliferation after infection, and the responses were efficiently maintained at long-term. The ability to induce cell-mediated immunity by all DNA vaccinated animals in presence or absence of CpG was considerably higher than mice receiving protein Ag in association with CpG. This enhanced cellular immune response obtained in DNA/DNA or DNA-prime/Protein-boost vaccination only because of stability exhibited by DNA-based vaccines [52].

It is fairly well established that IFN-γ and IL-12, the signature cytokines of Th1 responses, are decreased during active VL. Moreover, protective immunity against *L. donovani*, is dependent on an IL-12 driven Th1 response and IFN-γ production [53], [54] which results in the induction of parasite killing by macrophages via the production of reactive nitrogen and oxygen intermediates [55], [56]. However, cured VL patients displayed both Th1 and Th2 type clones producing IFN-γ, and IL-4 [57], control of *L. donovani* in susceptible BALB/c mice was associated with mixed Th1/Th2 responses [58], [59]. By contrast, there are reports of early mixed Th1/Th2 responses before infection and polarized Th1 responses after challenge infection leading protection against murine VL [32], [60]. Therefore, the existence of such a distinct Th1/Th2 polarization in VL is unclear. Hence, we analyze the cytokine profile before and after challenge infection in all vaccinated mice. IFN-γ production was elevated in gp63 DNA plus CpG vaccinated animals, while the responses was significantly enhanced after ten days and twelve weeks post-immunization in HPB regimen, before *L. donovani* challenge. Furthermore, co injection of CpG with either gp63 DNA or with rgp63 has a clear adjuvant effect in inducing IFN-γ in comparison to only gp63 DNA and rgp63 (data not shown). However, almost comparable level of IFN-γ was observed in mice receiving gp63 DNA alone and rgp63 plus CpG-ODN. Although, challenge with viable parasites enhanced IFN-γ production in all vaccinated mice except DNA-prime/Protein-boost regimen, the significant level of IFN-γ was still maintained in HPB regimen. Moreover, to analyze the interplay between CD4^+^ and CD8^+^ T cells in mediating IFN-γ production, *in vitro* blocking with either anti CD4^+^ Ab, or anti CD8^+^ Ab, or both was performed. In case of gp63 DNA vaccination, IFN-γ response was mainly mediated by CD8^+^ T cells, but CD4^+^ T cells also contribute to this response. On the other hand, in case of rgp63 plus CpG vaccination, IFN-γ was released mainly from CD4^+^ T cells, and partially from CD8^+^ T cells.

The mechanism of protective action of CpG-ODN against VL is correlated with the production of Th1 cytokine particularly, IL-12. Moreover, CpG-ODN 1826 is known to activate Langerhans cells, which in turn produce IL-12. Indeed endogenous IL-12 is required for clearance of parasites, the level of IL-12 was studied in all vaccinated mice. The levels was gradually increased in mice receiving either gp63 DNA alone, or in association with CpG, or in heterologously prime boost vaccination using CpG as adjuvant before and after challenge infection in both STP and LTP studies. Although, DNA-prime/Protein-boost showed significantly higher IL-12 (p40) responses, which was enhanced after *L. donovani* infection, lower IL-12 (p40) responses was obtained in mice receiving rgp63 plus CpG before and after challenge infection. Higher production of IFN-γ, and IL-12 produced by HPB regimen ultimately reflected towards highest nitrite production from cultured splenocytes.

Intriguingly, expression of an established Th2 cytokine like IL-4 was down-regulated after challenge infection in groups of mice receiving gp63 DNA mixed with CpG either homologously or heterologously. Since HPB regimen showed higher IL-4 after vaccination, and substantially lower IL-4 after challenge infection, early mixed Th1/Th2 responses exhibited by this groups of mice was therefore skewed in Th1 biased response after *L. donovani* infection. In addition, since the fact that IL-10 has been shown to block the Th1 activation and consequently a cytotoxic response by down regulating IL-12 and IFN-γ production, the disease associated macrophage deactivating cytokine was down-regulated significantly at twelve weeks post vaccination in mice vaccinated with HPB regimen.

To understand the disease outcome underlying these results, we therefore analyzed the parasite load in liver and spleen after *L. donovani* challenge infection. Even in the susceptible BALB/c mice, the gp63 DNA vaccination demonstrated potency and durability against experimental VL. However, data obtained through LDA suggest that DNA-prime/Protein-boost in presence of CpG led to almost 10^7^ fold and 10^10^ fold reduction in hepatic and splenic parasite burden in LTP study. This extent of protection, obtained in this study has till now not been achieved in long-lasting protection through HPB approach in susceptible BALB/c model against VL. DNA-prime/Protein-boost vaccination showed durable protection, which correlates with enhanced cellular and humoral responses before and after *L. donovani* challenge. More surprisingly, the course of *L. major* infection as measured by lesion development in the footpad suggested that mice vaccinated with DNA-prime/Protein-boost approach showed minor progress in lesion size, which also validates the protective role of this vaccine against murine CL.

Reasons for the enhanced efficacy of DNA vaccination over protein plus adjuvant may include low levels of persistent Ag, or presence of CpG motifs in their backbone [33]. Moreover, in this study, the enhanced efficacy shown by HPB regimen over DNA plus CpG is possibly due to the ability of the initial priming with DNA to prime T cells to generate elevated secondary responses or to produce high-affinity Ag-specific T cells whose numbers are increased following boosting with protein Ag.

In summary, our study demonstrated that DNA-prime/Protein-boost vaccination is effective in making durable vaccine against *Leishmania* infection in susceptible BALB/c mice. To our knowledge, this is the first detailed comparative study on protective efficacy and durability of DNA/DNA, DNA-prime/Protein-boost, Protein/Protein based vaccination against murine VL.
